# Intraoperative kidney replacement therapy in acute liver failure

**DOI:** 10.1007/s00467-023-06272-7

**Published:** 2024-03-25

**Authors:** Daniel Henderson, Anish Gupta, Shina Menon, Akash Deep

**Affiliations:** 1 Division of Liver Transplant, Anaesthetic Department, King’s College Hospital NHS Foundation Trust, Denmark Hill, London, UK; 2 Division of Nephrology, Department of Paediatrics, University of Washington and Seattle Children’s Hospital, Seattle, WA USA; 3 Paediatric Intensive Care Unit, King’s College Hospital NHS Foundation Trust, Denmark Hill, London, UK; 4 Department of Women and Children’s Health, School of Life Course Sciences, King’s College London, London, UK

**Keywords:** Intraoperative kidney replacement therapy, Acute liver failure, Children, Acute kidney injury, Liver transplantation, Paediatric intensive care

## Abstract

Paediatric acute liver failure (PALF) is often characterised by its rapidity of onset and potential for significant morbidity and even mortality. Patients often develop multiorgan dysfunction/failure, including severe acute kidney injury (AKI). Whilst the management of PALF focuses on complications of hepatic dysfunction, the associated kidney impairment can significantly affect patient outcomes. Severe AKI requiring continuous kidney replacement therapy (CKRT) is a common complication of both PALF and liver transplantation. In both scenarios, the need for CKRT is a poor prognostic indicator. In adults, AKI has been shown to complicate ALF in 25–50% of cases. In PALF, the incidence of AKI is often higher compared to other critically ill paediatric ICU populations, with reports of up to 40% in some observational studies. Furthermore, those presenting with AKI regularly have a more severe grade of PALF at presentation. Observational studies in the paediatric population corroborate this, though data are not as robust—mainly reflecting single-centre cohorts. Perioperative benefits of CKRT include helping to clear water-soluble toxins such as ammonia, balancing electrolytes, preventing fluid overload, and managing raised intracranial pressure. As liver transplantation often takes 6–10 h, it is proposed that these benefits could be extended to the intraoperative period, avoiding any hiatus. Intraoperative CKRT (IoCKRT) has been shown to be practicable, safe and may help sicker recipients tolerate the operation with outcomes analogous with less ill patients not requiring IoCKRT. Here, we provide a comprehensive guide describing the rationale, practicalities, and current evidence base surrounding IoCKRT during transplantation in the paediatric population.

## Introduction

Paediatric acute liver failure (PALF) is a condition often characterised by both its rapidity of onset and potential for significant morbidity and mortality. It can be seen in previously healthy children of all ages or as an acute decompensation in paediatric patients with chronic liver disease (CLD), paediatric acute on chronic liver failure (PACLF). Whilst some aetiologies mirror those of adult ALF, such as viral hepatitis and medication-related injury, congenital and metabolic causes are common in PALF. Furthermore, a much higher percentage of paediatric patients has an indeterminate cause of their ALF—of those children undergoing liver transplantation for PALF, almost 50% were linked to indeterminate causes [1]. Whilst the precise incidence in children is unknown, likely related to ongoing heterogeneity in diagnostic criteria (Table 1), it is estimated at 1–10 cases per million people per year across all age groups [2]. Approximately 20% of children with PALF undergo liver transplantation [2]. European datasets show that from 1968 to 2017, 16,641 paediatric liver transplants were undertaken on 14,515 patients at 133 centres [3]. PALF was the listed indication for transplant in 12% of cases. This proportion has stayed roughly stable over time. Congenital biliary disease (44%) and metabolic disease (18%) make up the most common causes for paediatric transplantation [3]. In the USA, the Paediatric Acute Liver Failure Study Group (PALFSG) in North America recruited 986 patients into their database over 11 years. National data also shows that PALF accounts for approximately 8–11% liver transplants in the USA [4–6].

**Table 1:** Definitions of paediatric acute and acute on chronic liver failure

**Paediatric acute liver failure** from the Paediatric Acute Liver Failure Study Group [7]:	**Paediatric acute on chronic liver failure** from the World Gastroenterology Organization [8]:
EITHER sudden onset of jaundice with evidence of liver aetiology	A syndrome characterised by acute hepatic decompensation resulting in liver failure (jaundice and prolongation of the INR) and *one or more extrahepatic organ failures* that is associated with increased mortality within a period of 28 days and up to 3 months from onset
OR incidental discovery of raised transaminases in association with symptoms suggesting liver injury of acute onset	
***Plus***	
INR > 2.0 due to liver dysfunction of less than 8 weeks duration with or without hepatic encephalopathy	
INR > 1.5 with encephalopathy	

From the above definitions, it is notable that encephalopathy is often salient to a diagnosis of acute liver failure [4, 9]. It is recognised that it is often more difficult to assess in children. Whilst similar scoring systems are used in both paediatric and adult populations, such as the West Haven criteria, these can be adapted to better reflect the subtle signs of encephalopathy in small children—particularly those non-verbal [4, 7, 9–12]

PALF often manifests in multiorgan dysfunction/failure characterised by prolonged periods of somewhat refractory haemodynamic instability, acid–base status imbalance, electrolyte abnormalities and impaired azotemic homeostasis. In their more extreme manifestations, these derangements can precipitate cerebral oedema. In the UK, the most severe cases of PALF may warrant performing a ‘super-urgent’ liver transplantation—this is transplantation in a patient who is not expected to survive more than 48 h without a transplant. This is a major surgical intervention which can worsen the difficulties already encountered in PALF. Even elective surgery, such as in living-related donor liver transplantation where typically there is more time to plan/optimise the recipient, adds additional complications such as substantial intraoperative blood loss alongside the resultant coagulopathy. Together, these difficulties frequently mandate the provision of organ support in an intensive care setting: inotropic/vasoactive support and large volume resuscitation using both fluid and transfused blood products.

Patients with PALF often require CKRT for management of AKI, fluid overload, refractory electrolyte, or acid base abnormalities [13, 14]. By providing clearance with gentle volume management, it helps maintain haemodynamic stability and act as a bridge to transplantation. Whilst large scale meta-analyses in adults have failed to show a survival benefit [15], it has been suggested that CKRT should be considered at an early stage to help prevent further deterioration and allow time for potential spontaneous recovery or bridge to liver transplantation [2, 14 16–19]. Whilst many of the indications for CKRT are resolved by liver transplantation, some patients have persistent fluid and electrolyte imbalances worsened by events in the operating room including haemodynamic instability, transfusion of large volumes of blood products, and effects of graft reperfusion. It has been posited that the use of IoCKRT during complex liver transplantation procedures may facilitate tolerance of these significant intraoperative shifts in volume alongside optimised acid–base and metabolic status. Together, these benefits should lessen their contribution to complications and adverse patient/graft outcomes.

Currently, published data regarding IoCKRT during liver transplantation has mostly been limited to case series and retrospective controlled cohorts [20–27]. Though not as robust as randomised controlled trials, these studies have suggested that IoCKRT was beneficial and, importantly, was not associated with increased rates of post-operative complications.

## AKI in PALF

The interaction between acute liver failure in children and kidney dysfunction can be complex and multifactorial. In PALF, the rapid decline in liver function can induce a systemic inflammatory response, leading to endotoxin and cytokine release. This response can result in haemodynamic changes, kidney vasoconstriction, hypoperfusion, and ultimately AKI. Moreover, direct hepato-kidney toxicity from accumulated metabolites such as bile acids, microcirculatory changes, aberrant coagulation, and oxidative stress also contribute to kidney damage [14, 16, 28]. The postulated mechanisms for AKI in PALF are highlighted in Table 2.

**Table 2:** Postulated mechanisms for kidney injury in PALF

**Kidney hypoperfusion:** reduced liver function can lead to decreased systemic vascular resistance and hypotension, which can result in kidney ischemia and subsequent AKI. Kidney perfusion may be further compromised by abdominal compartment syndrome due to organomegaly and ascites
**Hepatorenal syndrome:** functional kidney impairment that occurs as a consequence of severe liver dysfunction. The exact mechanisms underlying it are not fully understood but likely involve a combination of circulatory disturbances and changes in systemic and kidney haemodynamics
**Infection and sepsis:** paediatric patients with ALF are at increased risk of developing infections, which can progress to sepsis. Sepsis-associated AKI can occur due to a combination of hypoperfusion, direct bacterial invasion, and inflammatory responses
**Nephrotoxic medications:** some medications used in the management of ALF, such as certain antibiotics, can have nephrotoxic effects and contribute to the development of AKI

The significance of AKI in PALF can be understood in several ways:

1. Management: AKI complicates the management of PALF. It may necessitate alterations in fluid and electrolyte balance, adjustment of medication dosages, and initiation of kidney replacement therapy (such as haemodialysis or continuous kidney replacement therapy) to support kidney function whilst the liver recovers.
2. Liver transplantation: The presence of AKI can complicate the transplantation process, as it may affect the suitability of the patient for transplantation and impact post-transplant outcomes.
3. Prognosis: AKI is associated with poorer outcomes in patients with acute liver failure, including higher mortality rates. The presence and severity of AKI can serve as important prognostic indicators in determining the overall clinical course and outcome of PALF [17].

Whilst data specific to PALF are lacking, peri-transplantation AKI has been estimated to be present in 17.6 – 37.6% depending on the criteria used [29, 30]. Management of AKI in PALF aims to treat underlying causes, provide supportive care, and prevent further kidney damage. This includes aggressive management of infections, optimising fluid and electrolyte balance, correction of metabolic disturbances and avoiding nephrotoxic drugs. In severe cases, CKRT and/or liver transplantation may be necessary. Early recognition and intervention can potentially reverse AKI and improve outcomes. A multidisciplinary approach involving paediatric gastroenterologists, nephrologists, intensivists, and anaesthetists is key to managing these patients.

## Intraoperative continuous kidney replacement therapy

The concept of intraoperative continuous kidney replacement therapy (IoCKRT) is not novel in the adult population [21, 23, 26, 31]. However, it is much less reported in the paediatric population.

Whilst commencing KRT pre- and/or post-transplantation is more well established, continuing KRT intraoperatively is much less common. Opinions differ regarding the overall benefit of IoCKRT in liver transplant surgery, with limited evidence, particularly in the paediatric population [13, 28, 32].

Here, we consider the unique challenges that liver transplantation presents to the anaesthesia and PICU teams regarding KRT. The factors that influence the overall success of KRT intraoperatively are often different from those in the pre-/post-operative period. Firstly, we discuss the significantly altered, and potentially labile, haemodynamic changes that occur during various stages during the surgery and that play a huge role in the ability of the child to tolerate extracorporeal KRT. These are summarised in Table 3. Next, we consider the practicalities of maintaining the KRT machine in the operating theatre, the potential need for dialysate composition customisation and the importance of anticoagulation strategies considering significant surgical haemorrhage. The general and unique considerations needed for IoCKRT specifically are summarised in Table 4.

**Table 3:** Phases of liver transplant, associated haemodynamic changes and proposed benefits of IoCKRT

Phase of surgery	Cause of haemodynamic changes	Potential benefit of IoCKRT	Impact on patient and IoCKRT circuit	Strategies to mitigate impact on patients and circuit
Dissection of native liver	• Haemorrhage—increased in those with difficult anatomy or scarring due to previous surgery • Compressed/stretched central venous system during manipulation of liver affecting venous return to right ventricle • Toxic vasoactive mediators released causing vasodilatation	• Adjusting fluid removal during periods of bleeding • Stable metabolic/electrolyte background • Neuroprotection from optimised haemodynamics and clearance of toxins	• Hypotension; challenges with volume management • Need for transfusion of blood products	• Reduce or pause ultrafiltration • Consider using thromboelastography
Anhepatic	• Caval clamping leading to drop in central venous return to the heart producing hypotension and tachycardia	• Optimised potassium and acid–base balance whilst surgical haemostasis achieved	• Reduced venous return in patient and access alarms in circuit	• Reduce CKRT blood flow by 25–50% (and adjust RCA accordingly)
Reperfusion of the graft	• Caval clamp off: restores central venous return to the heart with increased cardiac output and potential surge in ICP • Portal clamp off: cold/ischaemic/hyperkaliaemic blood released—hypotension/arrhythmia	• Increased exchange rates to ameliorate the effects from reperfusion of portal system blood	• Potential for hyperkalaemia	• Consider transition to lower K dialysate/replacement fluids, when available, 30 min prior to unclamping • Frequent monitoring of K
Haemostasis, biliary anastomosis, closure	• Haemorrhage from cut-liver surface	• Optimised electrolyte and acid–base balance to promote optimised coagulation	• Hypotension; challenges with volume management	• Reduce or pause ultrafiltration

**Table 4:** A suggested prescription for IoCKRT for children undergoing liver transplantation

Vascular access	Internal jugular vein is preferred
Blood flow rate	Continue the same rate used pre-operatively or 3–8 mL/kg/min; may depend on the size of the patient, type of vascular access and the device used for IoCKRT
Modality	CVVH or CVVHD or CVVHDF may be done per institutional protocol
Clearance	3000–4000 mL/1.73 m^2^/h or 50–60 mL/kg/h Pre-operative clearance may be continued and adjusted based on intraoperative labs as needed
Dialysis/replacement fluid	Per institutional protocol; transition to lower potassium fluid may be done 30 min prior to unclamping of the IVC, where such fluids are available
Ultrafiltration rate	May be adjusted based on fluid balance and haemodynamics
Anticoagulation	Per institutional protocol; regional citrate anticoagulation, epoprostenol or nafamostat may be used safely without increasing the risk of bleeding

Broadly speaking, liver transplantation is classically divided into four phases:

- Dissection of the native liver
- Anhepatic phase
- Reperfusion of the graft
- Haemostasis, biliary anastomosis and closure

Dissection involves the initial subcostal incision and mobilisation of the native liver for removal. This is often the phase when the patient is at the highest risk of blood loss, particularly if this is prolonged due to complicated anatomy and/or presence of scar tissue and adhesions from previous surgery. Owing to the at times unpredictable nature of dissection, haemorrhage can be sudden and brisk making it difficult to keep pace with the blood loss. Given that even small volume haemorrhage often represents a much larger proportion of a child’s total blood volume, it is crucial to have increased vigilance to invasive monitoring and lab indices. These large volume shifts mean haemodynamics can be enormously labile. In addition to blood loss, mobilisation of structures and, in particular, lifting of the liver during dissection around the retro-hepatic region can significantly compress and stretch the central venous system adding to haemodynamic lability. Handling of the collapsed liver releases vasoactive mediators and toxins which can in turn cause hypotension and detrimental reductions in cerebral perfusion pressure (CPP). It can be postulated that IoCKRT should serve to help attenuate some of these haemodynamic difficulties by maintaining a stable metabolic and electrolyte milieu in the face of ongoing blood loss and subsequent transfusion. Also, in the presence of excessive haemorrhage, the volume of fluid removed can be adjusted to alleviate hypotension.

Patients with PALF are usually receiving higher clearance CKRT. Similar high clearance is continued intraoperatively to provide adequate toxin/cytokine and metabolite clearance due to the vast production by the toxic liver, coupled with the hyperdynamic vascular state. This dynamic process of toxin release, alongside ongoing haemorrhage, can exacerbate a developing coagulopathy and further worsen haemodynamics. Transfusion is more or less mandated in these cases with blood product transfusion strategies tailored to the individual case. Here, the use of point-of-care testing is particularly useful as opposed to relying on formal laboratory indices which can lag and become unrepresentative of the immediate situation. Thromboelastography, such as TEG™, is used to help guide product replacement in order to minimise exacerbations in coagulopathy by treating these early. However, it has been shown that transfusion rates have an influence on the development of post-op complications such as AKI in a dose-dependent manner [33, 34]. By helping to optimise the haemodynamics in the ways discussed, IoCKRT can further help to limit this coagulopathy and minimise the risk of over-transfusion.

The ability of IoCKRT to optimise haemodynamics, metabolic profiles and electrolyte balance is important individually. However, an amalgamation of these benefits can be used to explain a further potential role in neuroprotection.

Hepatic encephalopathy is important due to its association with intracranial hypertension and cerebral oedema which can result in spontaneous intracerebral haemorrhage. It is a strong predictor of mortality—hence its incorporation in King’s College criteria in the adult population.

**Fig. 1:**
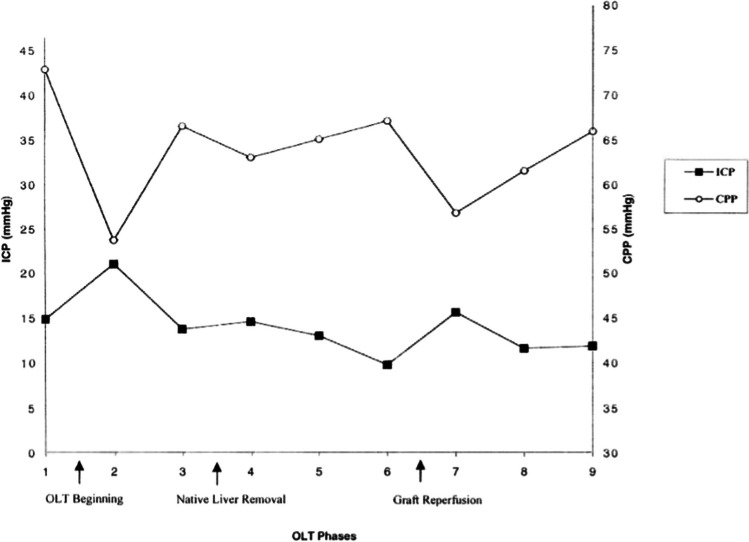
Intracranial pressure (ICP) and cerebral perfusion pressure (CPP) fluctuations during orthotopic liver transplant (OLT) surgery [36]

Furthermore, for those most severely affected and progressing towards liver transplant, it is known that the surgery itself can exacerbate neurological sequelae. Both dissection of the native/toxic liver and graft reperfusion phases are associated with raised intracranial pressure (ICP) and subsequently reduced CPP (see Fig. 1) [35].

Mechanisms postulated to contribute to HE/raised ICP include the following:

- Cytotoxicity from aberrant metabolic and electrolyte profiles—importantly hyperammonaemia due to deranged detoxification function of the liver to convert ammonia to urea which leads to astrocytic swelling due to osmotic effects of ammonia and glutamine
- Vasogenic oedema and blood-brain barrier (BBB) disruption
- Loss of cerebral blood flow (CBF) autoregulation

In summation, the autoregulatory vasomotor mechanisms promoting stability of CPP are hampered due to the multifaceted pathophysiological effects of active haemorrhage, manipulation of the central venous compartment, and the release of vasoactive mediators causing both systemic inflammation and microcirculatory alterations [37, 38]. The loss of this autoregulation means CPP is less able to compensate for changes in ICP promoting both cerebral hypoperfusion and hyperaemia, during different phases of the surgery. Ergo, the ability of IoCKRT to lessen/nullify some of these deleterious effects proposes further helpfulness in the role of neuroprotection.

During transition from dissection to anhepatic phase, cessation of blood supply to the failing liver, through tying the hepatic artery and portal vein, often leads to a marked improvement in haemodynamics since the release of toxins into the systemic circulation is reduced.

The anhepatic phase represents the time when all blood flow to and from the liver has ceased, having been carefully dissected off the vena cava. At this point, the liver is ready to be removed. A clamp is placed across the entire vena cava in close proximity to the diaphragm (sometimes pulling/stretching the right atrium), compromising venous return to the heart by as much as 30–40%. This is evidenced clinically by a drop in central venous pressure, blood pressure and compensatory tachycardia. It is crucial that the child is adequately filled prior to vena cava clamping or complete cardiovascular collapse may ensue. In reality, blood will often shunt via the azygos and hemiazygos venous system into the right heart, though less reliable in ALF as opposed to chronic portal hypertensive disease states.

Once the liver is removed, hypoglycaemia, acidosis and hypothermia will often worsen. Here, IoCKRT can help in temperature regulation through warming in the extracorporeal circuit. Depending on the CKRT machine, for very small children, the circuit lines themselves can be wrapped to insulate against heat loss during transit of blood from the warming device within the machine back to the patient. Some newer generation machines do a better job in temperature regulation than others [39, 40]. The anhepatic phase provides time for stabilisation and optimisation in preparation for reperfusion, where cerebral protection is the primary goal, and right heart strain is the main risk.

During this time, KRT should be adjusted to reflect any significant change in venous return on caval clamping and should aim to maintain acid–base balance now that the bleeding has been contained. Whilst anhepatic, the child may get severely acidotic with a huge rise in lactate. On approaching reperfusion, the foremost aim is to achieve a pH as normal as possible and to control the potassium in order to optimise cardiac output and haemodynamic competence. Dialysate composition may be manipulated here to use a lower potassium solution to create more resilience in the system. Intraoperative KRT techniques can be tailored to individual patient needs, allowing for precise adjustments in fluid management, solute clearance, and kidney support.

Reperfusion follows when the new liver’s blood vessels have been anastomosed to the child’s native vessels with reconnection of the grafted liver onto the vena cava and anastomosis of the portal vein. The initial release of the caval clamp primarily restores venous return to the right heart, increasing blood pressure and central venous pressure. However, once the portal venous clamp is removed, cold/acidotic/potassium-rich fluid directly hits the right heart and pulmonary vasculature. This has an unpredictable effect, often resulting in hypotension, arrhythmias and, in worse case scenarios, cardiac arrest. Surges in ICP are also possible on reperfusion and therefore, particularly in ALF, it is important that exchange rates are adequate to protect the brain.

Once the graft is reperfused via the portal vein, the surgeons will re-anastomose the hepatic artery. On removal of the hepatic artery clamp, a second stage reperfusion via the hepatic artery is seen—usually this is a much more benign reaction which uncommonly has any relevant sequalae. However, one should be mindful that occasionally a similar pattern is seen to that described with portal reperfusion.

The final stage involves obtaining surgical haemostasis, biliary reconnection, and finally abdominal closure. Since donated livers are often too large to transplant whole into children, grafts are usually cut down to an appropriate size prior to surgery. This means that grafts in children will have a cut-surface from which bleeding occurs and will need surgical control. Once adequate haemostasis has been achieved, the bile duct is either anastomosed onto the child’s bile duct (end to end) or a roux loop is created. Finally, the abdomen is closed either by primary closure or a temporising mesh is required.

IoCKRT requires collaboration between multiple teams including transplant surgery, nephrology, hepatology, anaesthesiology, and intensive care. It is important to have clear protocols in place, defining roles and responsibilities, lines of communication, and management plan (Table 5). General principles of CKRT hold true for IoCKRT as for pre-/post-op CKRT. Following on from reperfusion, generally the child is more settled, although haemodynamics typically take several days before returning to normal levels. It may then be possible to reduce the clearance from the initial higher levels, though this might be best done after a period of stability on PICU.

**Table 5:** Intraoperative CKRT checklist

Team
• Paediatric intensive care team
• Anaesthesiology
• Transplant surgeons
• Nephrologists
• CKRT nurse
• Operating room (OR) nurse
Supplies
• CKRT device for use
• Back-up CKRT device available (back-up device may be kept primed and ready per institutional protocol)
• Sufficient length of tubing for infusion pumps delivering calcium and citrate anticoagulation (ACD-A)
• Replacement and dialysate fluids, including lower potassium fluids if available
• Point-of-care device for ionised calcium monitoring
Intraoperative
• Reduce blood flow by 25–50% during IVC cross clamp
• Consider using lower potassium dialysis and replacement fluids, if available, 30 min prior to IVC unclamping
Postoperative
• Disconnect CKRT circuit
• Recirculate if planning to resume in post-operative period or discard circuit if not continuing
• Adjust dose based on laboratory parameters (particularly if patient was on higher clearance for hyperammonaemia)

## Prescription of IoCKRT

### Operating room logistics

Ideally, a walkthrough in the operating room (OR) with the anaesthesiologists, OR nurse, and CKRT specialists is recommended prior to the surgery to plan placement of CKRT device and allow vascath access without interfering with the sterile field. Where it has been instituted pre-operatively, CKRT may be paused for transfer. The machine can be placed in recirculation until the patient is ready for connection or a new circuit can be restarted in the OR. This avoids having to transfer the child to the OR connected to the CKRT device with the significant risk of vascath displacement. It can be restarted once the patient has been safely positioned for surgery and ergonomics of access lines and CKRT machines optimised. Some programs prefer to have a second identical CKRT device and circuit primed and ready for connection in case the first circuit clots.

### Vascular access

For liver transplant surgery, KRT is best performed via a vascath placed in the internal jugular vein. If either (right or left) internal jugular vein is not available, vascath may be placed in the subclavian vein. It is important to note that the incidence of thoracic central venous obstruction is 30–50% following catheters placed into the subclavian vein (not limited to haemodialysis catheters) [36]. Femoral vein is not suitable for IoCKRT due to clamping of the inferior vena cava (IVC) during liver transplantation, which can lead to access alarms for the CKRT system.

### Blood flow rate

This is usually kept at the same rate as pre-operative CKRT. During IVC cross clamp, the venous return and cardiac output can drop leading to access alarms. In such a situation, the blood flow rate may be decreased by 25–50%, along with a corresponding decrease in calcium and citrate infusions, if used for anticoagulation.

### CKRT modality and dose

The choice of CKRT modality (continuous veno-venous haemofiltration, CVVH; continuous veno-venous haemodialysis, CVVHD; or continuous veno-venous haemodiafiltration, CVVHDF) is centre-specific, and the modality used pre-operatively can be continued. Ammonia has a molecular weight of 17 daltons and is cleared well by both diffusive and convective modalities [41]. In one cohort of critically ill patients, small molecular (urea) clearance was similar during CVVH and CVVHD at a dose of 35 mL/kg/h although the filter life was better with CVVHD [42]. However, at the higher doses used in patients with PALF, CVVHD may be preferable as increasing the dialysate flow rate does not increase the filtration fraction as may be seen with high replacement flow rates.

The prescribed dose often depends on the pre-operative indication. Patients with PALF and hyperammonaemia are often started on a dose of 3000 mL/1.73 m^2^/h or 60 mL/kg/h, with the dose adjusted as needed based on clinical and laboratory profile. If the patient was receiving CKRT for AKI and/or fluid overload, without significant concern for hyperammonaemia, they may be on standard CKRT dose of 2000 mL/1.73 m^2^/h or 25–30 mL/kg/h. Patients who are receiving CKRT pre-operatively may be able to continue the same dose intraoperatively also, with adjustments made as needed based on labs. This dose may be delivered as replacement, dialysate, or a combination of both based on the modality used, CVVH, CVVHD, or CVVHDF.

### Dialysis/replacement fluid

The choice of replacement fluid may vary based on the device, local availability, and the phase of transplant surgery. Where low potassium dialysate/replacement fluids (solutions with 0, 1 or 2 mEq/L potassium) are available, centres may transition to using those about 30 min prior to unclamping of the IVC. Certain brands of CKRT fluids are only available with a potassium concentration of 4 mEq/L. In either of these situations, serum electrolytes should be checked every 30 min, dissection phase.

### Ultrafiltration

Often the patient is kept in a neutral balance to avoid large fluid shifts intraoperatively. During IVC unclamping, the risk of hypotension and haemodynamic instability are high, and ultrafiltration may need to be paused to avoid further compromise.

### Anticoagulation

This is often the same as pre-operative and depends on institutional practice. Heparin is typically avoided. Some adult programs use no anticoagulation. In paediatrics, regional citrate anticoagulation (RCA) and epoprostenol have been used safely for IoCKRT [43, 44].

RCA is typically preferred over heparin to avoid the risk of bleeding, particularly intraoperatively. However, in patients with liver failure, there is impaired citrate metabolism leading to the risk of citrate accumulation and toxicity which may manifest as hypocalcaemia and eventually metabolic acidosis. It has been used successfully in paediatric patients with liver failure, including intraoperatively [43, 45]. The citrate infusion in these patients is run at a lower rate than it would be for other patients receiving CKRT and a higher circuit ionised calcium may be tolerated (0.5–0.7) [45]. Whilst using RCA, the risk of additional citrate delivery from blood products and risk of citrate accumulation must be considered. Additionally, the calcium infusion for RCA must be clearly marked and should not be interrupted for any other use in case of limited vascular access.

Epoprostenol is the synthetic equivalent of prostacyclin which is a derivative of arachidonic acid and is produced by vascular endothelial cells [28]. Its anticoagulant effect is mediated by inhibition of platelet aggregation. Given its very short half-life of 2–8 min, it behaves almost like regional anticoagulation when infused into the circuit and has very few systemic adverse effects [28]. Side effects pertinent to intraoperative use include hypotension and ventilation-perfusion mismatch. Additionally, there may be increased incidence of bleeding in patients with ACLF and oesophageal varices. The usual dose range is 4–8 ng/kg/min.

Nafamostat mesylate, a synthetic serine protease inhibitor, may also be used, although there are no data on paediatric IoCKRT.

### Laboratory monitoring

The use of point-of-care testing is preferred over formal laboratory indices which may be delayed and not representative of the immediate situation. Thromboelastography, such as TEG™, can be used to help guide product replacement in order to minimise exacerbations in coagulopathy by treating these early. Patient ionised calcium and circuit ionised calcium required hourly if using RCA. Electrolytes are needed frequently around IVC unclamping.

### Termination or continuation of CKRT post-operatively

At end of the surgery, the nephrologist, transplant surgeon and intensivist make a shared decision to continue or terminate IoCKRT based on the patient’s clinical picture. If the therapy is terminated, the circuit is discontinued per institutional protocol. If CKRT is required post-operatively, the circuit may be recirculated and reinitiated after return to the ICU. The CKRT dose may need to be adjusted if the patient was previously receiving a higher clearance due to liver failure and hyperammonaemia.

## IoCKRT in children with chronic liver disease

As children with cirrhosis can have a haemodynamic picture not too dissimilar to the ALF cohort, many of the above principles may be applied to this group; especially in ACLF when a child with CLD slips into multiorgan failure due to an acute precipitating event like sepsis or bleeding varices. AKI in the setting of ACLF is a major determinant of mortality. CKRT is often initiated pre-operatively in these patients for fluid overload, AKI, metabolic abnormalities like hyperkalaemia, metabolic acidosis, hyperlactataemia.

One key difference between children with ALF and CLD is that these children with CLD have a venous system adapted to high portal pressures; they have opened collateral vessels to shunt venous blood into the systemic circulation. Therefore, when placing the caval clamp, they may be more readily able to compensate for hypotension by increasing their shunting and restoring more venous return from below the diaphragm.

Owing to the time course of CLD and the ability of the child to compensate for the more insidious pathophysiological processes, IoCKRT is generally not needed unless encountering near catastrophic complications or a child has severe pre-existing kidney disease with uncontrolled potassium levels prior to reperfusion. Some anaesthetists would recommend using IoCKRT if the child has been on pre-operative CKRT for a prolonged period due to complete kidney shutdown with a risk of further decompensation during the process of liver transplantation. This is especially likely in a child who has high vasopressor/inotrope requirements and/or unstable ventilation pressures and high oxygen demand (particularly related to fluid overload).

## IoCKRT in children with chronic non-cirrhotic liver disease

CKRT may be electively considered pre-transplant and during transplantation for some metabolic conditions. Often these children do not have a cirrhotic haemodynamic picture, but instead relatively normal systemic vascular resistance (SVR) and heart rate. Therefore, exchange rates need not be so high in this group. Some conditions include methyl malonic acidaemia and propionic acidaemia, particularly where these children have pre-existing kidney dysfunction.

Since these children do not have portal hypertension, they have not developed collaterals and are more prone to significant hypotension when the caval clamp is placed. KRT should be gentle to not exacerbate drops in venous return/hypotension.

## Future research topics

### Technology and innovation

Continued advancements in medical technology, including miniaturised dialysis machines, improved filtration techniques, and more precise monitoring tools, will further enhance the safety and efficacy of intraoperative RT.

### Personalised medicine

The integration of genetic and molecular profiling may help tailor intraoperative KRT to individual patient needs, optimising outcomes and minimising complications.

### Education and research

Further research and educational initiatives focusing on paediatric intraoperative KRT are essential to enhance understanding, refine techniques, and disseminate best practices globally.

### Data registry

Compile a detailed global registry of PALF based on agreed diagnostic criteria. This could include the incidence of AKI in these patients, which progress to commencement of KRT and longitudinal data surrounding ongoing kidney injury and the development of CKD.

## Conclusions

Considering AKI in PALF, understanding the underlying mechanisms, the need for timely diagnosis, and prompt management strategies are vital in optimising patient care and improving outcomes. The significance of AKI in PALF is consistently recognised regarding poorer outcomes for the graft and the patients overall.

CKRT has proven utility for critically ill children with PALF. It has been shown to improve haemodynamic and metabolic stability and can act as a bridge to liver transplantation. CKRT should be considered early in the management of PALF. Its continuation during surgery is both feasible and likely to confer better outcomes post-operatively. Importantly, intraoperative AKI management requires collaboration between stakeholders to overcome the various technical and logistical challenges.

Going forward, further research and collaborative efforts are necessary to explore novel therapeutic interventions and enhance our understanding of this complex interplay between liver and kidney function in paediatric patients with acute liver failure. Research should aim to clarify the optimal timing of commencement of CKRT and potential strategies to individualise therapies such as in dialysate composition and individualised CKRT protocols.

## Key summary points

- Acute liver failure in children is often associated with severe AKI.
- There is an increased morbidity and mortality risk in critically ill children who require CKRT for AKI and fluid overload.
- In those PALF patients undergoing liver transplantation, the surgery can be described in four main phases: dissection, anhepatic, reperfusion, haemostasis and biliary reconstruction.
- Intraoperative CKRT, whilst having its technical challenges, is likely to extend the benefits seen in pre-/post-operative CKRT to provide better graft and patient outcomes due to the ability to stabilise cerebral perfusion pressure by reducing haemodynamic variability.

## Multiple choice questions

Answers appear following the references.

1. What electrolyte abnormality is most likely to be seen around the time of IVC unclamping?
  - Hypokalaemia
  - Hyperkalaemia
  - Hyponatraemia
  - Hyperphosphataemia
2. Which anticoagulant is not recommended for intraoperative CKRT?
  - Nafamostat
  - Regional citrate
  - Heparin
  - Epoprostenol
3. What is/are the common indications for consideration of intraoperative CKRT?
  - Raised intracranial pressure
  - Severe AKI
  - Fluid overload
  - All of the above
4. Least preferred vascular access for IoCKRT in children:
  - Right internal jugular vein
  - Left internal jugular vein
  - Right femoral vein
  - Right subclavian vein
5. Considerations need to be given to the following during IoCKRT:
  - Trained staff to run CKRT intraoperatively
  - Staffing resources
  - Filter clotting during liver transplant
  - All of the above
